# Functional identification of genes responsible for the biosynthesis of 1-methoxy-indol-3-ylmethyl-glucosinolate in *Brassica rapa* ssp. *chinensis*

**DOI:** 10.1186/1471-2229-14-124

**Published:** 2014-05-08

**Authors:** Melanie Wiesner, Monika Schreiner, Rita Zrenner

**Affiliations:** 1 Leibniz-Institute of Vegetable and Ornamental Crops Grossbeeren and Erfurt e.V., Theodor-Echtermeyer-Weg 1, 14979 Grossbeeren, Germany

## Abstract

**Background:**

*Brassica* vegetables contain a class of secondary metabolites, the glucosinolates (GS), whose specific degradation products determine the characteristic flavor and smell. While some of the respective degradation products of particular GS are recognized as health promoting substances for humans, recent studies also show evidence that namely the 1-methoxy-indol-3-ylmethyl GS might be deleterious by forming characteristic DNA adducts. Therefore, a deeper knowledge of aspects involved in the biosynthesis of indole GS is crucial to design vegetables with an improved secondary metabolite profile.

**Results:**

Initially the leafy *Brassica* vegetable pak choi (*Brassica rapa* ssp. *chinensis*) was established as suitable tool to elicit very high concentrations of 1-methoxy-indol-3-ylmethyl GS by application of methyl jasmonate. Differentially expressed candidate genes were discovered in a comparative microarray analysis using the 2 × 104 K format *Brassica* Array and compared to available gene expression data from the *Arabidopsis* AtGenExpress effort. *Arabidopsis* knock out mutants of the respective candidate gene homologs were subjected to a comprehensive examination of their GS profiles and confirmed the exclusive involvement of polypeptide 4 of the cytochrome P450 monooxygenase subfamily CYP81F in 1-methoxy-indol-3-ylmethyl GS biosynthesis. Functional characterization of the two identified isoforms coding for CYP81F4 in the *Brassica rapa* genome was performed using expression analysis and heterologous complementation of the respective *Arabidopsis* mutant.

**Conclusions:**

Specific differences discovered in a comparative microarray and glucosinolate profiling analysis enables the functional attribution of *Brassica rapa* ssp. *chinensis* genes coding for polypeptide 4 of the cytochrome P450 monooxygenase subfamily CYP81F to their metabolic role in indole glucosinolate biosynthesis. These new identified *Brassica* genes will enable the development of genetic tools for breeding vegetables with improved GS composition in the near future.

## Background

**Figure 1:**
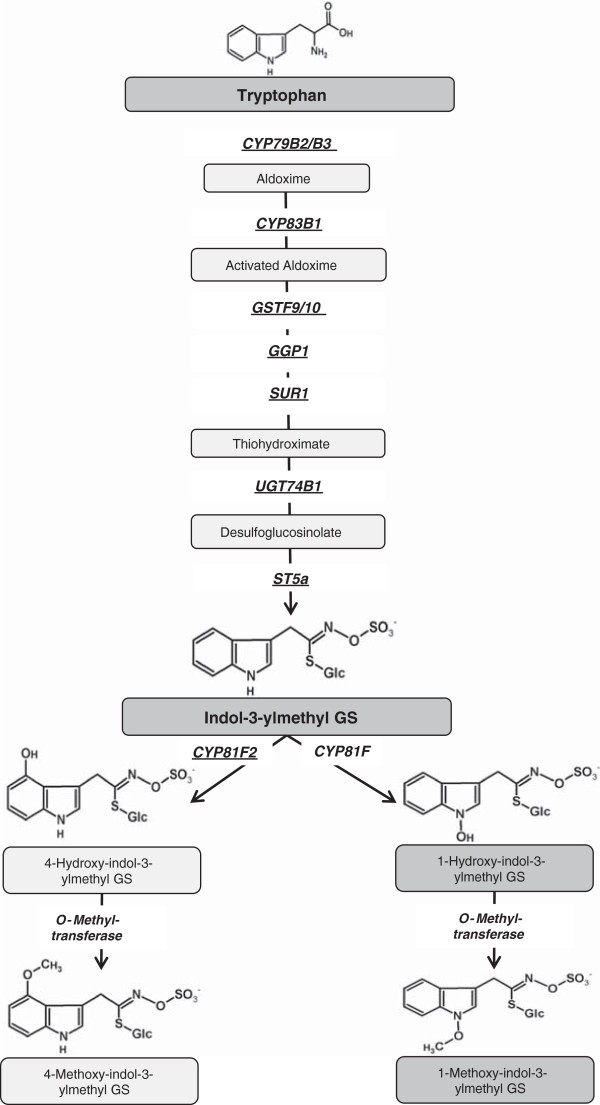
**Biosynthesis pathway of indole glucosinolates as known in *Arabidopsis thaliana*.** Enzymes catalyzing each reaction are given with the respective gene name. Identified putative *Brassica rapa* homologues [14] are indicated with underscores.

Glucosinolates (GS) are amino acid-derived nitrogen- and sulphur-containing plant secondary metabolites characteristic for most families of the order Brassicales [1,2]. Altogether there are about 200 known naturally occurring GS structures [3,4], of which various ecotypes of the model organism *Arabidopsis thaliana* have about 40 [5]. Depending on the amino acid precursor GS could be divided into three groups: (i) aliphatic GS derived from leucine, isoleucine, valine, and methionine; (ii) aromatic GS derived from phenylalanine and tyrosine; and (iii) indole GS derived from tryptophan. The biosynthesis of GS proceeds through three separate phases, the chain elongation of selected precursor amino acids, the formation of the core GS structure, and finally modifications of the side chain. Several genes of the biosynthetic network and key regulators for GS present in *Arabidopsis* are known [6,7]. The formation of the GS core structure is widely elucidated and genes responsible for secondary modifications of aliphatic GS via oxygenations, hydroxylations, alkenylations and benzoylations have been identified [8]. Indole GS can undergo hydroxylations and methoxylations, with CYP81F2 identified as the gene responsible for 4-hydroxylation of indol-3-ylmethyl GS (I3M) in *Arabidopsis*[9-11] (Figure 1), together with further members of the CYP81F family of *Arabidopsis thaliana* as being involved in 4-hydroxylation of indol-3-ylmethyl GS and/or 1-methoxy-indol-3-ylmethyl GS biosynthesis [12]. When tissue is damaged, the thioglucoside linkage of GS is hydrolyzed by myrosinases, enzymes that are spatially separated from GS in intact tissue. In the presence or absence of specifier proteins the degradation results in the formation of a variety of hydrolysis products [13].

The different groups of GS and their various degradation products are extensively studied metabolites. It has been shown that genes encoding enzymes of the specific glucosinolate biosynthesis pathways form stable co-expression clusters [15], and group together with tryptophan biosynthetic genes in response to stress conditions [16]. With respect to plant fitness they play important roles in plant defence against herbivores [17] and pathogens [9], and also abiotic stresses like UV-B irradiation specifically changes the GS profile [18]. In addition, there is increasing evidence that evolutionary and ecological forces shape polymorphism at loci involved in the GS-myrosinase defence system [19].

*Brassica* vegetables are cultivated and consumed worldwide and represent a highly important component in the human diet [20]. Their content of GS is varying dependent on genotype, development and environmental conditions [21] while the composition of GS and their respective degradation products is a major determinant of the characteristic flavor and smell of *Brassica* vegetables [22]. In addition, the secondary metabolites and their respective degradation products are believed to have protective cancer-preventing activity in higher animals and humans [23,24]. However, recent studies also provide evidence that juices of Brassicaceae might also be mutagenic because they form characteristic DNA adducts in bacteria and mammalian cells [25]. It is namely the 1-methoxy-indol-3-ylmethyl GS and its degradation products that have been shown to exert these negative effects [26,27].

With this study new genes where identified that are involved in the biosynthesis of indole GS, namely the synthesis of 1-methoxy-indol-3-ylmethyl GS with focus on *Brassica* vegetables. After establishing the leafy *Brassica* vegetable pak choi (*Brassica rapa* ssp. *chinensis*) as suitable tool to elicit very high concentrations of 1-methoxy-indol-3-ylmethyl GS by application of methyl jasmonate (MeJA) [28] the identification of genes involved in this process was possible by comparing expression pattern in pak choi using the 2 × 104 K format *Brassica* Array with publicly available gene expression data from the *Arabidopsis* AtGenExpress effort [29]. With the functional characterization of the identified genes new genetic tools for breeding healthy vegetables with improved GS composition will be possible in the near future.

## Results and discussion

### Increased indole GS biosynthesis in pak choi treated with methyl jasmonate

**Figure 2:**
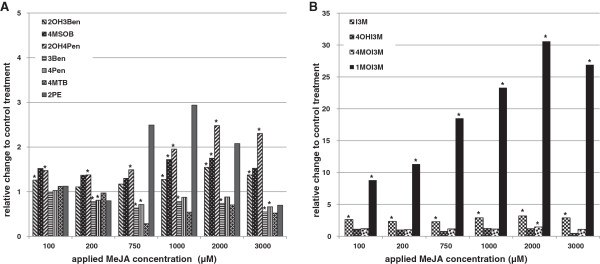
**Changes in the glucosinolate profiles in sprouts of pak choi (*Brassica rapa *ssp. *chinensis*) 48 hours after application of different concentrations of methyl jasmonate (MeJA). A**, relative changes to control of aliphatic and aromatic GS. **B**, relative changes to control of indole GS. 2OH3Ben, 2-hydroxy-3-butenyl GS; 4MSOB, 4-methylsulfinyl-butyl GS; 2OH4Pen, 2-hydroxy-4-pentenyl GS; 3Ben, 3-butenyl GS; 4Pen, 4-pentenyl GS; 4MTB, 4-methylthio-butyl GS; 2PE, 2-phenylethyl GS; I3M, indol-3-ylmethyl GS; 4OHI3M, 4-hydroxy-indol-3-ylmethyl GS; 4MOI3M, 4-methoxy-indol-3-ylmethyl GS; 1MOI3M, 1-methoxy-indol-3-ylmethyl GS. Values represent the mean of three independent samples. Significant differences to the respective control treatment (*P* < 0.05) as determined using unpaired two-tailed *t*-test, are marked with an asterisk. For absolute concentrations of glucosinolates please see supporting Additional file 1: Table S1.

In a previous study it was shown that different cultivars of the leafy vegetable pak choi (*Brassica rapa* ssp. *chinensis*) contain a certain amount of indole GS in their green leaf tissue [30]. The different cultivars can be classified in distinct groups depending on their GS profiles, which are partly linked to the expression of specific genes involved in the aliphatic GS biosynthetic pathway. In a related study it was further demonstrated that a small set of elicitors known to induce GS biosynthesis in various organism is also functional in pak choi [28]. Amongst others it was namely methyl jasmonate (MeJA) that led to an increase of indole GS biosynthesis. In order to further characterize this induction of GS biosynthesis in pak choi seedlings in more detail a concentration series ranging from 100 μM to 3 mM was applied and GS accumulation was measured 48 hours after application (Additional file 1: Table S1). As shown in Figure 2A a doubling of specific aliphatic GS could be achieved when applying concentrations of more than 750 μM MeJA, and also the amount of the aromatic 2-phenylethyl GS was increased up to 3fold at such high concentrations applied. As expected, indole GS accumulation was more sensitive to the MeJA application, and the indole GS level was elevated even when the lowest concentration of 100 μM was used (Figure 2B). With the application of higher concentrations of MeJA up to 2 mM a further increase of indole GS levels could be achieved until no additional elevation was detected. Notably it was mainly the 1-methoxy-indol-3-ylmethyl GS that was increased up to 30fold in pak choi seedlings after treatment with MeJA.

It is known for a long time that jasmonate, ethylene and salicylic acid upregulate the expression of scores of defense-related genes [31], and our knowledge of the complex network of jasmonate signaling in stress responses and development including hormone cross-talk is continuously increasing [32,33]. With respect to plant resistance GS present classical examples of compounds affecting insect-plant interactions [17] in which the GS-myrosinase defence system is also evolutionary and ecological modulated [19]. In terms of plants defense against pathogens it is further suggested that tryptophan-derived metabolites may act as active antifungal compounds [9,34]. Against this background the induced GS biosynthesis was strongly expected in pak choi after treatment with MeJA.

### Specific induction of 1-methoxy-indol-3-ylmethyl GS in pak choi seedlings

**Figure 3:**
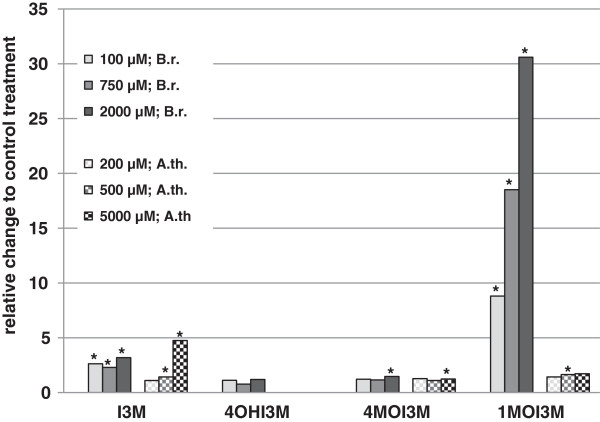
**Changes in the indole glucosinolate profiles of 12 day old seedlings.** Pak choi (*Brassica rapa* ssp. *chinensis*) (B.r.) and *Arabidopsis thaliana* Col-0 (A.th.) seedlings were treated with different concentrations of MeJA as indicated and glucosinolate profiles were determined 48 hours after application. B.r. treatment data are the same as in Figure 2; I3M, indol-3-ylmethyl GS; 4OHI3M, 4-hydroxy-indol-3-ylmethyl GS; 4MOI3M, 4-methoxy-indol-3-ylmethyl GS; 1MOI3M, 1-methoxy-indol-3-ylmethyl GS. 4OHI4M was undetectable in A.th. seedlings. Values represent the mean of three independent samples. Significant differences to the respective control treatment (*P* < 0.05) as determined using unpaired two-tailed *t*-test, are marked with an asterisk. For absolute concentrations of glucosinolates please see supporting Additional file 1: Table S2.

In order to analyze the specificity of the increased indole GS biosynthesis in more detail a similar experiment with *Arabidopsis* seedlings was performed using MeJA concentrations ranging from 200 μM up to 5 mM. As evident from Figure 3 MeJA application also increased indole GS content in *Arabidopsis* (Additional file 1: Table S2). However, the increase was much lower in this plant species, and the major elevation was found in the non-methoxylated indol-3-ylmethyl GS. Further experiments demonstrated that pak choi seedlings exert stronger rise of indole GS levels upon MeJA application than adult plants [28], while in *Arabidopsis* no differences in the elevation between seedlings and adult rosette leaves were detectable (data not shown). This comparison with *Arabidopsis thaliana* Col-0 ecotype clearly revealed that a very strong raise of 1-methoxy-indol-3-ylmethyl GS is specific to pak choi. The unambiguous difference between seedlings of pak choi and *Arabidopsis* discovered in this glucosinolate profiling analyses was used in further experiments to identify related genes involved in 1-methoxy-indol-3-ylmethyl GS biosynthesis of *Brassica rapa* ssp. *chinensis*.

### Identification of candidate genes using gene expression analysis with the Brassica microarray

As strong induction of 1-methoxy-indol-3-ylmethyl GS was found 48 hours after application of 2 mM MeJA to pak choi seedlings gene expression differences to control treatments were analyzed in these samples using the *Brassica* microarray. In order to get maximum amount of information the 2 × 104 K array was chosen in the investigation. The elements on the *Brassica* array were identified by their homology to known genes of *Arabidopsis thaliana* and were classified to respective bins using MapMan [35] and Mercator [36]. As expected when MeJA was applied to plant seedlings, defense related genes showed the most significantly changed transcript levels (Table 1). With respect to a putative function in GS metabolism [37] the genes with highest expression differences are listed in Table 2. Mainly the transcripts of genes putatively involved in GS degradation were induced, but also genes involved in indole GS core structure formation were strongly elevated and among the most significantly changed. The increased expression of genes specifically involved in indole GS core structure biosynthesis reflects the elevation of indole GS levels. Among the most significantly altered transcripts candidates were selected that are putatively involved in side chain modification of indole GS biosynthesis, namely those that show typical structures of the large gene families of cytochrome P450 monooxygenases or *O*-methyltransferases (Table 2).

**Table 1:** Expression differences in pak choi seedlings 48 hours after application of methyl jasmonate

**Identifier**	**Log2-fold change**	**Comparison with *Arabidopsis *** **sequences**
EV086532	8.3972	No similarity found
JCVI_8548	8.3041	Weakly similar to (164) AT1G72290| trypsin and protease inhibitor family protein/Kunitz family protein^a^
JCVI_27659	7.9237	Very weakly similar to (93.2) AT1G72290| trypsin and protease inhibitor family protein/Kunitz family protein^a^
EV175386	7.7622	No similarity found
JCVI_16491	7.6086	Moderately similar to (367) AT3G08860| alanine--glyoxylate aminotransferase, putative/beta-alanine-pyruvate aminotransferase
JCVI_3681	7.5634	Weakly similar to (176) AT1G73260| trypsin and protease inhibitor family protein/Kunitz family protein^a^
EV210392	7.4930	No similarity found
DW997085	7.4796	Moderately similar to (352) AT5G24420| glucosamine/galactosamine-6-phosphate isomerase-related
JCVI_25531	7.4141	Very weakly similar to (82.0) AT1G75940| ATA27 (A. thaliana anther 27); hydrolase, hydrolyzing O-glycosyl compounds
JCVI_3301	7.4066	Moderately similar to (292) AT5G07470| PMSR3 (PEPTIDEMETHIONINE SULFOXIDE REDUCTASE 3)^a^
JCVI_38382	7.2248	Moderately similar to (374) AT1G54040| TASTY, ESP (EPITHIOSPECIFIER PROTEIN)^b^
JCVI_20214	6.7805	Weakly similar to (199) AT3G12500| PR3, PR-3, CHI-B, B-CHI, ATHCHIB (BASIC CHITINASE); chitinase^a^
EV022852	6.6726	No similarity found
JCVI_19372	6.6380	Moderately similar to (272) AT3G55970| oxidoreductase, 2OG-Fe(II) oxygenase family protein
JCVI_11797	6.5346	Highly similar to (577) AT2G39310| jacalin lectin family protein^a^
EE568322	6.5096	Weakly similar to (124) AT3G08860| alanine--glyoxylate aminotransferase, putative/beta-alanine-pyruvate aminotransferase
JCVI_2201	6.3891	Weakly similar to (189) AT1G73260| trypsin and protease inhibitor family protein/Kunitz family protein^a^
EX126494	6.3312	Weakly similar to (152) AT1G66700| PXMT1; S-adenosylmethionine-dependent methyltransferase
JCVI_19562	6.3230	Weakly similar to (104) AT2G43510| ATTI1 (ARABIDOPSIS THALIANA TRYPSIN INHIBITOR PROTEIN 1)^a^
CD833129	6.1070	Weakly similar to (118) AT1G47540| trypsin inhibitor, putative^a^
EX037239	6.1057	No similarity found
JCVI_342	6.0609	Moderately similar to (240) AT1G72290| trypsin and protease inhibitor family protein/Kunitz family protein^a^
EE451932	6.0344	Very weakly similar to (87.8) AT3G08860| alanine--glyoxylate aminotransferase, putative/beta-alanine-pyruvate aminotransferase
JCVI_40366	5.9683	Moderately similar to (435) AT4G03070| AOP1 (2-oxoglutarate dependent dioxygenase 1.1); oxidoreductase
JCVI_8581	5.9431	Moderately similar to (349) AT1G52400| BGL1 (BETA-GLUCOSIDASE HOMOLOG 1); hydrolase^a^
JCVI_7526	5.9123	Moderately similar to (442) AT1G52400| BGL1 (BETA-GLUCOSIDASE HOMOLOG 1); hydrolase^a^
JCVI_37097	5.7525	Moderately similar to (309) AT1G66700| PXMT1; S-adenosylmethionine-dependent methyltransferase
JCVI_3160	5.5469	Weakly similar to (178) AT4G29270| acid phosphatase class B family protein
CX191896	5.5195	No similarity found
EX133344	5.4854	Moderately similar to (392) AT1G07440| tropinone reductase, putative/tropine dehydrogenase
EX037465	5.4674	Weakly similar to (123) AT3G49360| glucosamine/galactosamine-6-phosphate isomerase family protein
JCVI_22700	5.4095	Weakly similar to (196) AT5G59490| haloacid dehalogenase-like hydrolase family protein
JCVI_7218	5.4086	Moderately similar to (291) AT4G37410| CYP81F4 (cytochrome P450, family 81, subfamily F, polypeptide 4); oxygen binding^b^
EV124048	5.3916	Weakly similar to (128) AT4G35160| O-methyltransferase family 2 protein^b^
JCVI_31414	5.2952	Weakly similar to (191) AT4G29710| phosphodiesterase/nucleotide pyrophosphatase-related
EV125432	5.2734	Moderately similar to (240) AT4G37410| CYP81F4 (cytochrome P450, family 81, subfamily F, polypeptide 4); oxygen binding^b^
JCVI_33618	5.2687	Moderately similar to (457) AT4G35160| O-methyltransferase family 2 protein^b^
H74959	5.2324	No similarity found
JCVI_15025	5.2252	Moderately similar to (311) AT3G12520| SULTR4;2 (sulfate transporter 4;2); sulfate transmembrane transporter^b^
EX039068	5.1985	Weakly similar to (110) AT4G31500| SUR2, RNT1, ATR4, CYP83B1 (CYTOCHROME P450 MONOOXYGENASE 83B1); oxygen binding^b^
EX083822	5.1636	Very weakly similar to (91.7) AT1G54040| TASTY, ESP (EPITHIOSPECIFIER PROTEIN)^b^
CV432816	5.1395	Moderately similar to (320) AT1G66700| PXMT1; S-adenosylmethionine-dependent methyltransferase
JCVI_22851	5.1432	Moderately similar to (255) AT5G06860| PGIP1 (POLYGALACTURONASE INHIBITING PROTEIN 1); protein binding
JCVI_7995	5.0811	Moderately similar to (393) AT3G60140| SRG2, DIN2 (DARK INDUCIBLE 2); hydrolase
EX117993	4.9563	Moderately similar to (414) AT5G04380| S-adenosyl-L-methionine:carboxyl methyltransferase family protein
ES906294	4.8431	Moderately similar to (293) AT1G62660| beta-fructosidase (BFRUCT3)/beta-fructofuranosidase/invertase, vacuolar
CV433026	4.8167	Very weakly similar to (80.5) AT3G45140| ATLOX2, LOX2 (LIPOXYGENASE 2)^a^
JCVI_19710	4.8088	Moderately similar to (314) AT3G45140| ATLOX2, LOX2 (LIPOXYGENASE 2)^a^
EV209435	4.7071	No similarity found
JCVI_14756	4.7010	Moderately similar to (319) AT3G08860| alanine--glyoxylate aminotransferase, putative/beta-alanine-pyruvate aminotransferase

The *Brassica* 95 K unigene set was compared to *Arabidopsis thaliana* TAIR9 genome release and mapped to MapMan bins. Respective *Brassica* identifiers are shown, and relative changes to controls are given as log2-ratios. Grading of sequence similarity scores of the comparison with *Arabidopsis* sequences is as follows: highly similar, 501–1000; moderately similar, 201–500; weakly similar, 101–200. a, stress related, MapMan BinCode20; b, sulfur assimilation and glucosinolate metabolism, MapMan.

**Table 2:** Selected expression differences in pak choi seedlings 48 hours after application of methyl jasmonate

	**Identifier**	**Log2-fold change**	**Comparison with *Arabidopsis *** **sequences**
**Glucosinolate metabolism**	JCVI_38382	7.225	Moderately similar to At1g54040, ESP, epithiospecifier protein
	EX039068	5.199	Weakly similar to At4g31500, SUR2, CYP83B1, chytochrom P450 monooxygenase 83B1
	JCVI_24334	4.326	Highly similar to At2g22330, CYP79B3, cytochrome P450 monooxygenase 79B3
	JCVI_41905	4.265	Moderately similar to At4g39940, AKN2, APS-kinase 2
	JCVI_10889	4.238	Moderately similar to At5g14200, 3-isopropylmalate dehydrogenase
	JCVI_10648	3.943	Moderately similar to At4g39940, AKN2, APS-kinase 2
	JCVI_1353	3.140	Moderately similar to At1g54020, myrosinase-associated protein
	JCVI_16379	3.055	Highly similar to At4g39950, CYP79B2, cytochrome P450 monooxygenase 79B2
	JCVI_33391	2.466	Highly similar to At4g39950, CYP79B2, cytochrome P450 monooxygenase 79B2
	EV159250	2.317	Weakly similar to At1g52040, MBP1, myrosinase-binding protein 1
	JCVI_2556	2.299	Weakly similar to At1g52030, MBP2, myrosinase-binding protein 2
	JCVI_109	2.151	Moderately similar to At4g31500, SUR2, CYP83B1, chytochrom P450 monooxygenase 83B1
	JCVI_31290	2.117	Moderately similar to At1g24100, UGT74B1 UDP-glucosyl transferase 74B1
	JCVI_15640	1.969	Weakly similar to At1g62540, flavin-containing monooxygenase family protein
	JCVI_3890	1.953	Moderately similar to At5g25980, TGG2, glucoside glucohydrolase 2
**Candidate genes**	JCVI_7218	5.409	Moderately similar to At4g37410, CYP81F4, cytochrome P450 monooxygenase 81F4
	EV124048	5.392	Weakly similar to At4g35160, *O*-methyltransferase family protein
	EV125432	5.273	Moderately similar to At4g37410, CYP81F4, cytochrome P450 monooxygenase 81F4
	JCVI_33618	5.269	Moderatey similar to At4g35160, *O*-methyltransferase family 2 protein
	JCVI_40877	4.207	Moderately similar to At4g37430, CYP81F1, CYP91A2, cytochrome P450 monooxygenase 81F1
	JCVI_39399	3.658	Moderately similar to At1g13080, CYP71B2, cytochrome P450 monooxygenase 71B2
	JCVI_12863	3.217	Weakly similar to At5g42590, CYP71A16, cytochrome P450 monooxygenase 71A16
	EV170929	2.549	Moderately similar to At3g28740, CYP81D11, cytochrome P450 monooxygenase 81D11
	JCVI_8990	1.808	Moderately similar to At5g36220, CYP91A1, CYP81D1, cytochrome P450 monooxygenase

The *Brassica* 95 K unigene set was compared to *Arabidopsis thaliana* TAIR9 genome release and mapped to MapMan bins. Respective *Brassica* identifiers are shown, and relative changes to controls are given as log2-ratios. Grading of sequence similarity scores of the comparison with *Arabidopsis* sequences is as follows: highly similar, 501–1000; moderately similar, 201–500; weakly similar, 101–200. Genes with significantly altered expression and similarity to *Arabidopsis* genes with function in GS metabolism and genes with significantly altered expression and similarity to candidate genes of the gene families of cytochrome P450 monooxygenases and *O*-methyltransferasese are listed.

These selected candidates were further evaluated regarding respective expression differences of the related homologs in available *Arabidopsis thaliana* microarray hybridization experiments using the Genevestigator database [38]. As shown in Table 3 the *Arabidopsis* homologs of the selected genes involved in GS metabolism were found responsive to MeJA treatments with log2-ratios being 1 or greater. This is in good agreement with the reported modulation of the GS profile in *Arabidopsis* by defense signaling pathways [39] and is also reflected in results presented in Figure 3. The *Arabidopsis* homologs of the selected candidate genes show strong variation in their responsiveness to MeJA. While *At3g28740* (CYP81D11) and *At5g36220* (CYP81D1) were strongly induced by MeJA application, *At4g37410* (CYP81F4), *At4g37430* (CYP81F1) and *At5g42590* (CYP71A16) were only weakly influenced, while *At4g35160* (OMT) and *At1g13080* (CYP71B2) showed unchanged expression. As *At1g13080*, *At5g42590* and *At3g28740* were already expected to be involved in other metabolic pathways we concentrate in further experiments on *At4g37410* and *At4g37430* as genes putatively involved in GS metabolism, and on *At4g35160* and *At5g36220* without any linked pathway identified so far.

**Table 3:** **Evaluation of expression differences upon methyl jasmonate application of *Arabidopsis thaliana *** **genes involved in glucosinolate metabolism and respective homologs of candidate genes**

***Arabidopsis *** **gene, encoded protein**	**Induction**	**Related pathway**	***Brassica *** **identifier**
*At1g54040*, ESP, epithiospecifier protein	++	GS degradation	JCVI_38382
*At4g31500*, SUR2, CYP83B1, chytochrom P450 monooxygenase 83B1	++	GS biosynthesis	EX039068
			JCVI_109
*At2g22330*, CYP79B3, cytochrome P450 monooxygenase 79B3	++	GS biosynthesis	JCVI_24334
*At4g39940,* AKN2, APS-kinase 2	++	Sulfur assimilation	JCVI_41905
			JCVI_10648
*At5g14200*, 3-isopropylmalate dehydrogenase	++	GS biosynthesis	JCVI_10889
*At1g54020*, myrosinase-associated protein	+++	GS degradation	JCVI_1353
*At4g39950*, CYP79B2, cytochrome P450 monooxygenase 79B2	++	GS biosynthesis	JCVI_16379
			JCVI_33391
*At1g52040*, MBP1, myrosinase-binding protein 1	+++	GS degradation	EV159250
*At1g52030*, MBP2, myrosinase-binding protein 2	+++	GS degradation	JCVI_2556
*At1g24100*, UGT74B1 UDP-glucosyl transferase 74B1	++	GS biosynthesis	JCVI_31290
*At1g62540*, flavin-containing monooxygenase family protein	++	GS biosynthesis	JCVI_15640
*At5g25980*, TGG2, glucoside glucohydrolase 2	++	GS degradation	JCVI_3890
**Homologs of candidate genes, encoded proteins**			
*At4g37410*, CYP81F4, cytochrome P450 monooxygenase 81F4	+	Putative GS metabolism	JCVI_7218
			EV125432
*At4g35160*, *O*-methyltransferase family 2 protein	0	Unknown	EV124048
*At4g37430*, CYP81F1, CYP91A2, cytochrome P450 monooxygenase 81F1	+	Putative GS metabolism	JCVI_40877
*At1g13080*, CYP71B2, cytochrome P450 monooxygenase 71B2	0	Putative amino acids and derivatives	JCVI_39399
*At5g42590*, CYP71A16, cytochrome P450 monooxygenase 71A16	+	Put. triterpene, sterole, brassinosteroide	JCVI_12863
*At3g28740*, CYP81D11, cytochrome P450 monooxygenase 81D11	+++	Putative phenylpro-panoid metabolism	EV170929
*At5g36220*, CYP91A1, CYP81D1, cytochrome P450 monooxygenase	+++	Unknown	JCVI_8990

The Genevestigator database [37] was used to evaluate expression differences of the selected genes. *Arabidopsis* genes homologous to the identified MeJA responsive *Brassica* genes are in the same order as in Table 2. Grading of changes of the log2-ratios is as follows: 0, unchanged with log2-ratio smaller 0.5; +, log2-ratio between 0.5 and 1; ++, log2-ratio between 1 and 2.5; +++, log2-ratio larger than 2.5.

### GS profiling in *Arabidopsis* mutants with knock out of the respective candidate gene homologs

In order to verify a putative involvement of the selected candidate genes in indole GS biosynthesis respective *Arabidopsis* knock out mutants were profiled for their GS accumulation. Since there are tissue specific differences in the proportional distribution of individual GS with indole GS being mainly present in either roots or old leaves [40] plants were grown in tissue culture and leaves and roots analyzed separately, or GS profiles of leaves of flowering plants grown in the greenhouse were measured (Additional file 1: Table S3). As evident from Table 4 there is one of the four selected *Arabidopsis* knock out mutants that did not produce 1-methoxy-indol-3-ylmethyl GS in any of the tissues analyzed. This confirms the expectation that the *Arabidopsis* gene product of *At4g37410* (CYP81F4) is needed in leaves and roots to synthesize 1-methoxy-indol-3-ylmethyl GS [12,41]. The absence of a metabolic phenotype on GS level in the selected *Arabidopsis* mutant with knock out in the selected *O*-methyltransferase (*Atomt*) further shows that at least in *Arabidopsis* there are other *O*-methyltransferases present which could contribute to the synthesis of 1-methoxy-indol-3-ylmethyl GS in leaves. Consequently, it needs to be analyzed whether the *O*-methyltransferase activity is provided through IGMT5 (*At1g76790*) an *O*-methyltransferase family protein that is strongly co-expressed with *At4g37410* (CYP81F4) as determined using the ATTED-II coexpression database [42]. In addition, in *Arabidopsis* there are further members of the *O*-methyltransferase family, IGMT1 (*At1g21100*), IGMT2 (*At1g21120*) and IGMT4 (*At1g21130*), that are coexpressed with *At5g57220* (AtCYP81F2). At least in an artificial expression system using *Nicotiana benthamiana* it has been shown that IGMT1 and IGMT2 can be employed for *O*-methylation of indole GS [12].

**Table 4:** **Glucosinolate content in different tissues of selected *Arabidopsis *** **mutants**

**Mutant (gene)**	**Tissue**	**3MSOP**	**4MSOB**	**4MTB**	**5MSOP**	**8MSOO**	**I3M**	**4MOI3M**	**1MOI3M**	**Total GS**
***Atcyp81f4*** (At4g37410)	Leaves	92 ± 20	90 ± 14	97 ± 11	104 ± 11	102 ± 19	108 ± 11	98 ± 21	-*	95 ± 11
	Roots	-	-	-	-	98 ± 15	163 ± 15*	120 ± 21	-*	113 ± 13
***Atcyp81f1*** (At4g37430)	Leaves	99 ± 3	92 ± 1	123 ± 14	102 ± 4	96 ± 5	129 ± 17	99 ± 6	103 ± 2	104 ± 2
	Roots	-	-	-	-	93 ± 1	104 ± 2	99 ± 11	79 ± 15	89 ± 2
***Atcyp81d1*** (At5g36220)	Leaves	101 ± 9	108 ± 1	81 ± 4	98 ± 21	122 ± 16	87 ± 4	101 ± 5	97 ± 31	96 ± 2
	Roots	-	-	-	-	108 ± 5	96 ± 2	101 ± 7	169 ± 36	112 ± 8
***Atomt*** (At4g35160)	Leaves of flowering plant	76 ± 30	103 ± 43	-	-	-	97 ± 76	7 ± 2*	40 ± 15*	90 ± 47

3MSOP, 3-methylsulfinyl-propyl GS; 4MSOB, 4-methylsulfinyl-butyl GS; 4MTB, 4-methylthio-butyl GS; 5MSOP, 5-methylsulfinyl-pentyl GS; 8MSOO, 8-methylsulfinyl-octyl GS; I3M, indol-3-ylmethyl GS; 4MOI3M, 4-methoxy-indol-3-ylmethyl GS; 1MOI3M, 1-methoxy-indol-3-ylmethyl GS. Values represent the mean ± standard deviation of three to six individual plants homozygous for the respective T-DNA insertion. Significant differences to the respective control tissue (*P* < 0.05) as determined using unpaired two-tailed *t*-test, are marked with an asterisk. Values are given in % on dry matter basis of the respective control tissue. For absolute concentrations of glucosinolates in the respective control tissue please see supporting Additional file 1: Table S3. -, below detection limit.

As shown previously there is a certain increase of indole GS biosynthesis in *Arabidopsis* after application of MeJA (Figure 3). Therefore, the selected knocks out mutants of genes responsive to MeJA treatment (Table 3) were also analyzed after application of this elicitor. While mutants in AtCYP81F1 and AtCYP81D1 showed a comparable increase of indole GS biosynthesis as the treated control plants (Table 5), the mutant in AtCYP81F4 did not accumulate any 1-methoxy-indol-3-ylmethyl GS while an expected increase of the precursor indol-3-ylmethyl GS could be observed 48 hours after MeJA application in this mutant. This finally confirms that the gene product of *At4g37410*, the cytochrome P450 monooxygenase 81F4 is utterly necessary to synthesize 1-methoxy-indole-3-ylmethyl GS in *Arabidopsis* at standard growth conditions. It additionally demonstrates that there is none of the other P450 monooxygenase 81F family proteins involved in 1-methoxy-indole-3-ylmethyl GS synthesis even under conditions of increased biosynthesis when defense related pathways are induced*.*

**Table 5:** **Glucosinolate content in *Arabidopsis *** **mutants 48 hours after application of methyl jasmonate**

**Mutant**	**Treatment**	**Total aliphatic GS**	**I3M**	**4MOI3M**	**1MOI3M**	**Total indol GS**	**Total GS**
***Atcyp81f4***	500 μM MeJA	71 ± 21	86 ± 30	162 ± 12*	-*	87 ± 23	75 ± 21
***Atcyp81f1***	500 μM MeJA	83 ± 10	52 ± 5*	257 ± 17*	110 ± 10	81 ± 5	83 ± 7
***Atcyp81d1***	200 μM MeJA	86 ± 30	98 ± 6	72 ± 6	98 ± 50	95 ± 5	86 ± 26

Total aliphatic GS, 3-methylsulfinyl-propyl GS; 4-methylsulfinyl-butyl GS; 4-methylthio-butyl GS; 5-methylsulfinyl-pentyl GS; 8-methylsulfinyl-octyl GS. Total indol GS, I3M, indol-3-ylmethyl GS; 4MOI3M, 4-methoxy-indol-3-ylmethyl GS; 1MOI3M, 1-methoxy-indol-3-ylmethyl GS. Values represent the mean ± standard deviation of three individual plants homozygous for the respective T-DNA insertion. Significant differences to the respective control treatment (*P* < 0.05) as determined using unpaired two-tailed *t*-test, are marked with an asterisk. Values are given in% on dry matter basis of the respective treatment of control plants. -, below detection limit in mutant.

### *Arabidopsis* ecotype Wu-0 without 1-methoxy-indol-3-ylmethyl GS accumulation

Further evidence of the importance of *At4g37410* (CYP81F4) for 1-methoxy-indol-3-ylmethyl GS biosynthesis is coming from a survey of the GS content in leaves and roots of the 19 key accessions [43] used to develop the MAGIC lines [44]. A total of 20 distinct GS could be identified and quantified by Witzel and co-workers, with most of the aliphatic GS showing accession-specific distribution while the indole GS were present in almost all 19 accessions [43] with one exception: ecotype Wu-0 did not contain 1-methoxy-indol-3-ylmethyl GS in any tissue analyzed. Since the corresponding whole genome sequences of all 19 accessions are available [45] the respective sequence variants at locus *At4g37410* (http://mus.well.ox.ac.uk/19genomes/variants.tables/) were inspected for the presence of relevant polymorphisms. Indeed, at bp coordinate 18595917 in the pseudo genome and bp coordinate 17592444 of the Col-0 reference genome on chromosome 4 the insertion of one C nucleotide could be found solely in the accession Wu-0. This insertion produces a frame shift in the coding sequence thus disrupting CYP81F4 and leading to an altered protein sequence from amino acid 390 with a premature stop at amino acid 395. In contrast, the putative functional protein is composed of 501 amino acids in all other accessions that produce 1-methoxy-indole-3-ylmethyl GS. In summary this is an excellent example were publicly available sequence data together with comprehensive metabolite profiling enables the identification of a gene that is putatively involved in the respective metabolic pathway at question. In addition, since the ecotype Wu-0 is an *Arabidopsis* accession collected from Germany the presence of 1-methoxy-indol-3-ylmethyl GS does not seem to be essential for survival of this ecotype in its natural habitat. As shown previously defense related co-expression networks in *Arabidopsis thaliana* group together with tryptophan and GS biosynthesis genes in response to stress conditions [16]. Thus, the increase of indole GS biosynthesis in *Arabidopsis* and the relatively small accumulation of 1-methoxy-indol-3-ylmethyl GS when compared to *Brassica rapa* ssp. *chinensis* revealed that this specific indole GS might not play a pivotal role in stress response in *Arabidopsis thaliana*.

### Characterization of the CYP81F4 genes identified in the *Brassica rapa* genome

It was already shown that genes involved in the GS biosynthesis exist in more than one copy in the *Brassica rapa* genome accession Chiifu-401-42 [37]. Besides this there is also a high co-linearity when compared to *Arabidopsis thaliana.* This co-linearity is similarly found for AtCYP81F4 (*At4g37410*) surrounded by AtCYP81F3 (*At4g37400*) and AtCYP81F1 (*At4g37430*) on *Arabidopsis* chromosome 4. When compared to *Arabidopsis At4g37410* two different orthologues of the *Brassica rapa* accession Chiifu-401-42 on BAC clones KBrB006J12 and KBrH064I20 could be identified: While KBrB006J12 corresponds to a region on chromosome A01, no match for KBrH064I20 has been found so far. On KBrB006J12 the orthologue to AtCYP81F4 was identified as Bra011759 (BrCYP81F4-1) on the reverse strand on chromosome A01, and is preceded by Bra011758 orthologous to AtCYP81F3 and followed by Bra011761 orthologous to AtCYPF1. On KBrH064I20 the orthologue to AtCYP81F4 was named BrCYP81F4-2, and is preceded by another orthologue to AtCYP81F3 while the following sequence orthologous to AtCYPF1 is corrupted.

In order to analyze the tissue specific expression of the selected genes in more detail isoform specific primer pairs were developed using the respective sequences of the *Brassica rapa* accession Chiifu-401-42 BAC clones KBrB006J12 and KBrH064I20. Semi-quantitative realtime RT-PCR analysis was performed with cDNA synthesized from RNA isolated from 12 days old seedlings, and leaves and roots of six weeks old *Brassica rapa* ssp. *chinensis* plants. As evident from Table 6 expression of all selected genes could be detected in pak choi. In most cases a higher expression was found in leaves than in seedlings and only *BrCYP81F4-1* is expressed at a higher level in roots than in leaves. The highest expression level in leaves was detected for *BrCYP81F4-2* while *BrCYP81F4-1* was the main expressed isoform in roots. This already indicates that the *BrCYP81F4* isoforms may play an important role on a tissue-specific level and during development at standard growth conditions.

**Table 6:** Semi-quantitative realtime RT-PCR analysis of the selected genes in different tissues of pak choi

**Abbreviation**	**Treatment**	**Seedlings**	**Leaves**	**Roots**
***BrCYP81F1***	Control	-9.2 ± 0.64	-3.6 ± 0.68	-11.6 ± 2.76
	500 μM MeJA	-7.4 ± 0.18	-4.2 ± 2.05	-12.1 ± 1.52
***BrCYP81F2***	Control	-10.6 ± 0.37	-6.8 ± 1.26	-9.1 ± 0.94
	500 μM MeJA	-7.7 ± 0.42	-6.9 ± 1.43	-8.2 ± 0.75
***BrCYP81F3-1***	Control	-6.3 ± 0.23	-3.4 ± 0.48	-6.5 ± 0.57
	500 μM MeJA	-5.7 ± 0.42	-3.2 ± 0.44	-6.9 ± 0.45
***BrCYP81F3-2***	Control	-6.5 ± 0.20	-6.4 ± 0.74	-8.9 ± 0.32
	500 μM MeJA	-5.7 ± 0.20	-5.2 ± 1.41	-6.6 ± 0.41
***BrCYP81F4-1***	Control	-7.5 ± 0.61	-3.5 ± 0.30	-2.1 ± 0.30
	500 μM MeJA	-2.4 ± 0.65	0.3 ± 0.96	1.5 ± 0.20
***BrCYP81F4-2***	Control	-8.5 ± 0.40	-2.3 ± 0.37	-6.2 ± 0.60
	500 μM MeJA	-0.7 ± 0.75	2.3 ± 0.52	1.4 ± 0.60
***BrOMT***	Control	-8.3 ± 0.51	-5.5 ± 0.81	-6.7 ± 0.91
	500 μM MeJA	-3.0 ± 0.99	nd	nd

Each value represents the Ct value relative to that of *Actin* and is given as mean ± standard deviation of four individual samples. Measurements were repeated twice. Methyl jasmonate (MeJA) treatment was done 48 hours before harvest. nd, not determined.

**Figure 4:**
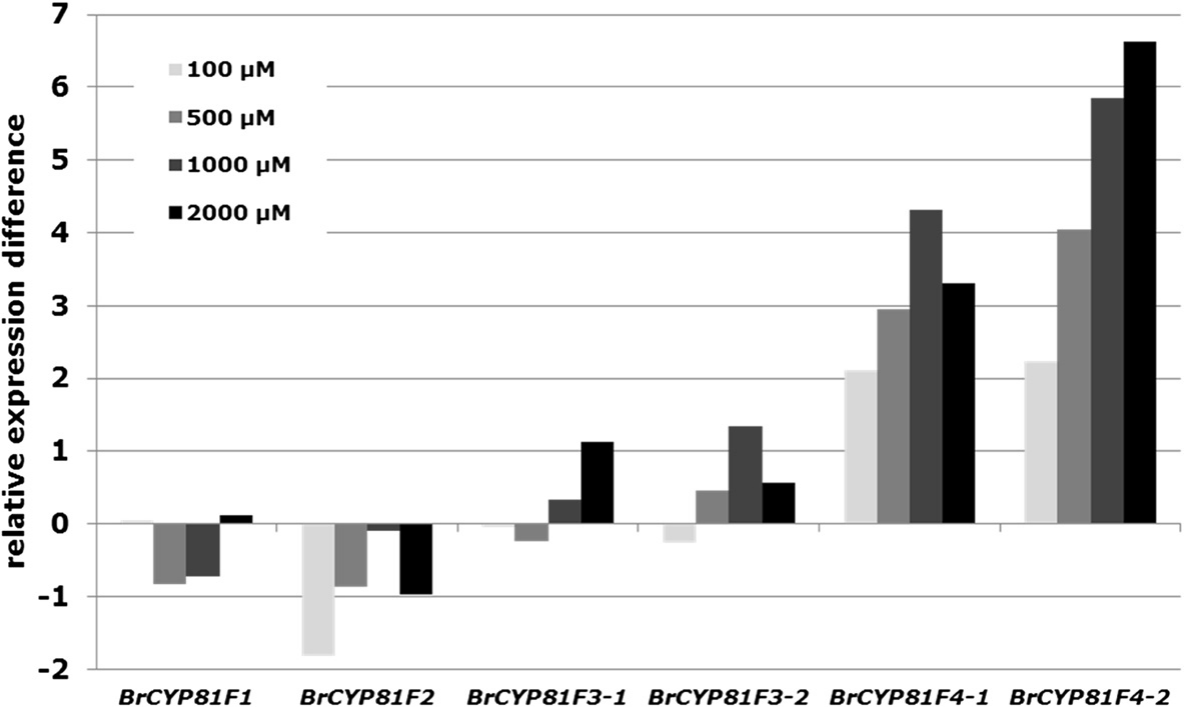
**Semi-quantitative realtime RT-PCR analysis of BrCYP81F genes in seedlings of pak choi (*Brassica rapa *ssp. *chinensis*) 48 hours after application of different concentrations of methyl jasmonate (MeJA).** Values represent the difference of the Ct value relative to that of Actin. Each value represents the mean of nine individual samples. Measurements were repeated twice. Relative expression differences to the control treatment are shown (ΔΔCt).

Further expression analysis was performed with different tissues of pak choi treated with 500 μM MeJA. Expression analysis confirmed induction of mainly the two identified *BrCYP81F4* genes in *Brassica rapa* ssp. *chinensis* seedlings, leaves and roots treated with MeJA (Table 6). Since there was some increased expression also detectable for other isoforms seedlings of pak choi were treated with a series of different concentrations of MeJA and expression differences to control treatment were analyzed for all *BrCYP81F* (Figure 4). This unequivocally confirms that both *BrCYP81F4* isoforms were most responsive to the elicitor treatment while the others did not show comparable sensitivity to this elicitor. Application of 100 μM MeJA already elevated the expression of *BrCYP81F4-1* and *BrCYP81F4-2* 4fold with highest increase of *BrCYP81F4-2* of more than 64fold after application of 2 mM MeJA. This confirms that the two isoforms of *BrCYP81F4* are the candidate genes from *Brassica rapa* ssp. *chinensis* that are crucial for 1-methoxy-indol-3-ylmethyl GS biosynthesis.

Jasmonic acid signaling is a central component of inducible plant defense and the expression of jasmonate-induced responses are tightly regulated by the ecological background of the plant [46] and also by the plant species itself. While in *Arabidopsis thaliana* tryptophan and GS biosynthesis genes respond to stress conditions [16] there is only relatively small accumulation of 1-methoxy-indol-3-ylmethyl GS when compared to *Brassica rapa* ssp. *chinensis*. The role of this distinct response to the elicitor and differences in accumulation of a specific defense compound will be the subject of future analysis in an ecological context.

### Functional identification of BrCYP81F4 isoforms for biosynthesis of 1-methoxy-indol-3-ylmethyl GS

In order to finally assess BrCYP81F4 isoform function full length cDNAs of both genes were amplified and heterologously expressed in the *Arabidopsis thaliana* mutant *Atcyp81f4*, which does not produce 1-methoxy-indol-3-ylmethyl GS. Using oligonucleotide primers developed with the *Brassica* A genome sequence from *Brassica rapa* accession Chiifu-401-42 [37] two full length cDNA sequences from *Brassica rapa* ssp. *chinensis* coding for putative BrCYP81F4 isoforms were amplified. Both sequences show 90.7% pair-wise identities and code for proteins of 501 amino acids with 93% similarity. Compared to the *Arabidopsis* protein similarities of 85.4% and 90.2% could be calculated. The sequences of interest (*BrCYP81F4-1* and *BrCYP81F4-2*) were recombined into the plant expression vector pK7WG2 [47] and *Agrobacterium* mediated gene transfer was performed using the knock out mutant *Atcyp81f4* as the host. Kanamycin resistant seedlings of the T2 generation were selected and analyzed for heterologous gene expression and GS accumulation. As shown in Table 7 expression of both cDNAs from pak choi in the *Atcyp81f4* mutant background led to metabolic complementation with accumulation of 1-methoxy-indol-3-ylmethyl GS in leaves and a reduced level of I3M when compared to the mutant without expression of the *Brassica rapa* ssp. *chinensis* genes. Although the identical heterologous expression system was used, BrCYP81F4-2 led to much higher accumulation of 1-methoxy-indol-3-ylmethyl GS. Whether this difference is caused by a higher protein level of the heterologous enzyme in the mutant plant background or is linked to advanced enzyme activity will be the topic of further studies. Another interesting point here is the significant decrease of I3M in the *Atcyp81f4* mutant background when the highly active BrCYP81F4-2 is expressed. In summary the level of indole GS stayed constant in these plants demonstrating unaltered total flux into the indole GS pathway thus indicating no further metabolic regulation by the end products.

**Table 7:** **Glucosinolate profiles in leaves of *Arabidopsis *** **mutants *Atcyp81f4 *** **transformed with the respective expression vector constructs**

**Mutant lines**	**Expression construct**	**3MSOP**	**4MSOB**	**4MTB**	**5MSOP**	**8MSOO**	**4OHI3M**	**I3M**	**4MOI3M**	**1MOI3M**
M3-1	Control	0.92 ± 0.08	6.26 ± 0.96	0.93 ± 0.17	0.16 ± 0.02	0.44 ± 0.08	0.05 ± 0.01	1.40 ± 0.19	0.37 ± 0.04	0.00 ± 0.00
M3-6	Control	0.90 ± 0.22	6.22 ± 1.54	0.89 ± 0.37	0.18 ± 0.05	0.50 ± 0.14	0.04 ± 0.01	1.25 ± 0.23	0.34 ± 0.04	0.00 ± 0.00
M3-1	35S::BrCYP81F4-1	0.90 ± 0.18	6.16 ± 1.54	0.80 ± 0.11	0.17 ± 0.04	0.42 ± 0.12	0.04 ± 0.01	1.21 ± 0.16	0.32 ± 0.04	0.08 ± 0.01*
M3-6	35S::BrCYP81F4-1	0.99 ± 0.03	7.67 ± 0.62	0.64 ± 0.09	0.21 ± 0.02	0.42 ± 0.07	0.02 ± 0.01	1.59 ± 0.22	0.34 ± 0.06	0.09 ± 0.03*
M3-1	35S::BrCYP81F4-2	0.86 ± 0.07	6.47 ± 1.07	1.01 ± 0.61	0.19 ± 0.04	0.66 ± 0.41	0.00 ± 0.00*	0.64 ± 0.35*	0.27 ± 0.01*	0.97 ± 0.64*
M3-6	35S::BrCYP81F4-2	0.67 ± 0.13	4.71 ± 0.90	0.57 ± 0.05	0.13 ± 0.03	0.28 ± 0.06*	0.01 ± 0.01*	0.43 ± 0.09*	0.20 ± 0.04*	0.86 ± 0.40*

3MSOP, 3-methylsulfinyl-propyl GS; 4MSOB, 4-methylsulfinyl-butyl GS; 4MTB, 4-methylthio-butyl GS; 5MSOP, 5-methylsulfinyl-pentyl GS; 8MSOO, 8-methylsulfinyl-octyl GS; 4OHI3M, 4-hydroxy-indol-3-ylmethyl GS; I3M, indol-3-ylmethyl GS; 4MOI3M, 4-methoxy-indol-3-ylmethyl GS; 1MOI3M, 1-methoxy-indol-3-ylmethyl GS. Values represent the mean ± standard deviation of three to six individual plants homozygous for the respective T-DNA insertion and transformed with the respective expression constructs. Significant differences to respective control tissue (*P* < 0.05) as determined using unpaired two-tailed *t*-test, are marked with an asterisk. Values are given in μmol * g^-1^ dry weight. Control, vector control pK7WG2; 35S::BrCYP81F4-1, expression construct using destination vector pK7WG2 recombined with BrCYP81F4-1; 35S::BrCYP81F4-2, expression construct using destination vector pK7WG2 recombined with BrCYP81F4-2.

## Conclusions

In conclusion this is an explicit example were elicitation of a specific metabolic difference and subsequent comparative microarray analysis together with focused metabolite profiling permits the targeted discovery of genes involved in the respective metabolic pathway. Here this enables the functional attribution of new identified *Brassica rapa* ssp. *chinensis* genes to their metabolic role in indole glucosinolate biosynthesis that in the near future will contribute to develop new genetic tools for breeding vegetables with improved glucosinolate profile.

## Methods

### Plant material

Seeds of *Brassica rapa* ssp. *chinensis* (pak choi) cultivar Black Behi (Allied Botanical, Quezon City, Philippines) were sown on bars of fleece, 3 g seeds of pak choi, placed in aluminum foil trays (33 × 10 cm) filled with perlite. Trays were kept in a greenhouse chamber at 12 h photoperiod (220 μmol m^-2^ s^-1^ of photosynthetic active radiation) and temperature regime of 24/20°C (day/night) at relative humidity about 75% for 10 days. The seedlings were watered as needed, no fertilizer was added. To obtain soil grown plants seedlings were germinated and grown on soil at 10 h photoperiod (photon flux density 150 μmol m^-2^ s^-1^, 22°C light, 20°C dark).

*Arabidopsis thaliana* L. Heynh Columbia-0 (Col-0), SALK_024438 (*Atcyp81f4*), SALK_031939 (*Atcyp81f1*), SALK_005073C (*Atcyp81d1*), and SALK_053994 (*Atomt*) were obtained from Nottingham Arabidopsis Stock Centre (University of Nottingham, Loughborough, United Kingdom). Seeds were surface sterilized and aseptically grown on ½ strength MS medium including vitamins [48], 0.5% sucrose and 0.7% agar. For elicitor treatment 20 mg of Col-0 seeds were spread per petri dish and grown in a greenhouse at 16 h photoperiod (photon flux density 250 μmol m^-2^ s^-1^) for 10 days. In all other cases seeds were imbibed at 4°C darkness (48 h) and grown in 10 h photoperiod (photon flux density 150 μmol m^-2^ s^-1^, 21°C). To obtain soil grown plants seedlings were transferred after three weeks to soil at 10 h photoperiod (photon flux density 150 μmol m^-2^ s^-1^, 22°C light, 20°C dark).

### Elicitor treatment

Methyl jasmonate (Sigma Aldrich, Seelze, Germany) was resolved in water containing 0.01% (v/v) Tween20 to reduce surface tension and water containing 0.01% (v/v) Tween20 was sprayed as control treatment. The 10 days old pak choi seedlings were treated by spraying each bar of fleece with 15 ml of the respective solution. The 10 days old *Arabidopsis* seedlings were treated by spraying each petri dish with 2 ml of the respective solution. 48 hours after treatment the total aerial tissue was harvested. Samples were quickly frozen in liquid nitrogen, subsequently lyophilized, and blended to a fine powder. For each treatment, at least three samples were taken as replicates.

### Sample preparation and desulfo-glucosinolate analysis by HPLC

Glucosinolate concentration was determined as desulfo-glucosinolates according to Wiesner et al. [30]. Briefly, 20 mg of powdered samples were extracted and analyzed by HPLC using a Merck HPLC system (Merck-Hitachi, Darmstadt, Germany) with a Spherisorb ODS2 column (Bischoff, Leonberg Germany; particle size 5 μm, 250 mm × 4 mm). Desulfo-glucosinolates were identified based on comparison of retention times and UV absorption spectra with those of known standards. Glucosinolate concentration was calculated by the peak area relative to the area of the internal standard. Each replicate sample was measured in duplicate. Results are given as μmol g^-1^ dry weight.

### Microarray analysis

The microarray analysis was performed as described [18]. Briefly, frozen pak choi sprout material was ground in liquid nitrogen in an orbital ball mill for 2 min at a frequency of 30 Hz s^-1^ (MM400 Retsch GmbH, Haan, Germany). Total RNA was extracted using the RNeasy Plant Mini Kit (Qiagen GmbH, Hilden, Germany), including the on-column DNase digestion step with the RNase-free DNase Set (Qiagen). The microarray analysis was done with 1 mg of total RNA isolated from each of three replicates of methyl jasmonate treated and control treated seedlings. Agilent One-Color Gene Expression Microarray analysis following the recommendation of MIAME (http://www.mged.org) was performed at Beckman Coulter Genomics (Morrisville, NC, United States, http://www.beckmangenomics.com/) using the 2 × 104 k format *Brassica* Array [49]; http://brassica.bbsrc.ac.uk/). Microarray data are available in the ArrayExpress database (http://www.ebi.ac.uk/arrayexpress) under accession number E-MTAB-2386. The Open Source Microarray Processing Software Robin (http://mapman.gabipd.org/web/guest/home) was used to evaluate and calculate results of the log fold change of expression in MeJA treated seedlings in relation to the control [35]. The assignment of the different genes was done by comparison of the translated protein sequences of the 95 k *Brassica* unigene set with the *Arabidopsis* TAIR9 database using the Mercator pipeline for automated sequence annotation [36] (http://mapman.gabipd.org/web/guest/app/mercator). For each identifier the gene with the highest homology was provided with identifier and description. The respective bitscores were classified as follows: very weakly similar (bitscore smaller than 100); weakly similar (bitscore 101–200); moderately similar (bitscore 201–500); highly similar (bitscore greater than 500).

### Isolation of mutants

Plants were obtained from the Salk collection [50]. Screening and selection within mutant populations was done following the Signal Salk instructions (http://signal.salk.edu). Genomic DNA was isolated by a standard procedure using NucleoSpin PlantII (Macherey-Nagel GmbH & Co. KG, Dueren, Germany). PCR genotyping was performed using the T-DNA LB-specific primer SALK LBb 5′-GCGTGGACCGCTTGCTGCAACT and the gene-specific primer pairs of Atcyp81f4l2 5′- AGGGTATTCGTTTTGGAGCA, Atcyp81f4r2 5′- CTTCTCCACCGTTGAACCTC; Atcyp81f1l2 5′- CTCCAACGAAAGCAACGATT, Atcyp81f1r2 5′- CGAGCATCATCGACTTCACA; Atcyp81d1l 5′- TGCCCATTCTAGAGTGACTGC, Atcyp81d1r 5′- AGAATGATGACCGGAAAACG; Atomtl 5′- CAAGTATTCCCATCGTCTCTCC, Atomtr 5′- ATTGAAAACCATCCTTCGTCAC. Homozygous mutants were isolated from selfed populations of the respective mutant. Gene knock-out was proven by semi-quantitative realtime RT-PCR.

### Gene expression analysis by semi-quantitative realtime RT-PCR

RNA was extracted from 100 mg tissue using the NucleoSpin Plant Kit (Macherey-Nagel GmbH and Co KG), including on-column DNaseI digestion. RNA was quantified spectrophotometrically at 260 nm (Nanodrop ND1000, Technology Inc., USA), and quality was checked using the ratio of absorption at 260 and 280 nm with a ratio between 1.9 and 2.1 as acceptable. Single-stranded cDNA synthesis was carried out with total RNA using SuperScript™ III RNaseH–reverse transcriptase (Invitrogen, Life Technologies GmbH, Darmstadt, Germany) with oligo d(T_12–18_) primers according to the manufacturer’s instructions. PCR amplified sequences generated with these oligonucleotide primer pairs and cDNA from pak choi leaves as template were subcloned and verified by sequence analysis. Semi-quantitative two-step RT-PCR was performed using a SYBR® Green 1 protocol in 96-well reaction plates on an Applied Biosystems 7500 Realtime PCR System. The following thermal profile was used for all reactions: 50°C for 2 min, 95°C for 10 min, 40 cycles of 95°C for 30 s and 60°C for 1 min, followed by dsDNA melting curve analysis to ensure amplicon specificity. Each reaction was done in a 10 μl volume containing 200 nM of each primer, 3 μl of cDNA (1:50) and 7 μl of Power SYBR Green Master Mix (Applied Biosystems, Life Technologies, Carlsbad, CA, USA). Data generated by semi-quantitative real-time PCR were collected and compiled using 7500 v2.0.1 software (Applied Biosystems). Data were exported to LinReg software [51] to determine the PCR amplification efficiency for each primer pair. Relative transcript levels of *Arabidopsis thaliana* were normalized on the basis of expression of *At3g18780* (*ACT2*), and relative transcript levels of *Brassica rapa* were normalized on the basis of expression of an invariant control orthologous to *At3g18780* on KBrB071H12 by calculating Δ*Ct*, the difference between control and target products (Δ*Ct* = *Ct*_gene_–*Ct*_act_)[52]. Semi-quantitative PCR was performed on at least three biological replicates measured in duplicates for each gene, and non-template controls were included. Gene-specific primer sets are listed in Table 8.

**Table 8:** Oligonucleotide primers used for gene expression analysis

**Oligonucleotide abbreviation**	**Sequence**	**Accession (Gene abbreviation)**
At-ACT2f	TCCCTCAGCACATTCCAGCAGAT	At3g18780
At-ACT2r	AACGATTCCTGGACCTGCCTCATC	(AtACT2)
At-CYP81F1f	TACTGAGAAATCCAGAAGTACT	At4g37430
At-CYP81F1r	GTTTTGGAGGTAAGGAAGCAC	(AtCYP81F1)
At-CYP81F4f	TTGTTGAACCACCCAAAAGTTT	At4g37410
At-CYP81F4r	GGAGGTAAGGAAGGTTTGCT	(AtCYP81F4)
At-MT2f	CCGGCTTGCGACGCCATTT	At4g35160
At-MT2r	TTTTATTCTCTCCGATCACCGAT	(AtOMT)
At-CYP81D1f	TGCTTAACCATCCTGACGTAA	At5g36220
At-CYP81D1r	CTTTAGATATGGTAGCTCGCTA	AtCYP81D1)
BrAf	ACGTGGACATCAGGAAGGAC	AC189447
BrBr	CTTGGTGCAAGTGCTGTGAT	(BrACT2)
BrCYP81F1f	TCCCTCGCACGCCGACG	KBrB006J12.9 Bra011761
BrCYP81F1r	AGGATGCGGCAGCGAGTTA	(BrCYP81F1)
BrCYP81F2f	TCTCCTTCTGAAGATCTCAAAA	KBrB027E01.6 Bra006830
BrCYP81F2r	GTGTTCGCTGCTTCTTTTTCT	(BrCYP81F2)
BrCYP81F3f1	GCCGAGATCACCGATGGAA	KBrB006J12.6 Bra011758
BrCYP81F3r1	TGAACGTCTTCTCCTCCGC	(BrCYP81F3-1)
BrCYP81F3f2	GCCAAGATCGACGACAGAC	KBrH064I20.2
BrCYP81F3r2	GTCTTCTCCTCCTTCTCCGA	(BrCYP81F3-2)
BrCYP81F4f1	TTAACGGAAGAGGACATCAAAG	KBrB006J12.7 Bra011759
BrCYP81F4r1	AAAGAGGGGAAGGAGACAAAGA	(BrCYP81F4-1)
BrCYP81F4f2	TTAACAGTAGAGGACATCAAGA	KBrH064I20.1
BrCYP81F4r2	TGGAGGAGAAGGAGAAAAGGA	(BrCYP81F4-2)
BrOMTf1	GGCTGTACCGGAGAGACGA	Bra017700
BrOMTr1	GCCGTTCTCATCAAGTGGGTG	(BrOMT)

### Cloning procedures and plant transformation

All constructs have been made using a combination of TOPO® and GATEWAY® cloning system (Invitrogen). *Brassica rapa* subsp. *chinensis* sequences coding for the two identified, putative CYP81F4 were amplified using the Advantage® 2 PCR Kit (Clontech, Takara Bio Company, Kyoto, Japan) and primer pairs BrF4-1fg 5′- CACCATGTTCTACTATGTGATACTCCCT and BrF4-1ro 5′- AACCTTTGAGTCGGTAACAA; as well as BrF4-2fg 5′- CACCATGTTTTACTATGTGATTCTCCCT and BrF4-2ro 5′- AACTTTTGACTCGGTAAGAA. PCR products were inserted into the entry vector pENTR™/SD/D-TOPO® (Invitrogen), and verified by sequencing (LGC Genomics GmbH, Berlin, Germany). Both sequences of interest (*BrCYP81F4-1* (Accession KF612589) and *BrCYP81F4-2* (Accession KF612590)) were then recombined into the appropriate destination vector pK7WG2 [47] using GATEWAY® LR Clonase™ II enzyme mix according to the manufactures instructions (Invitrogen). *Agrobacterium* mediated gene transfer was performed according to [53] using two homozygous lines (M3-1, M3-6) of the knock out mutant *Atcyp81f4* as the host. Kanamycin resistant seedlings of the T1 generation were selected and expression of the respective transgene was recorded by semi-quantitative realtime RT-PCR.

## Competing interests

The authors declare that they have no competing interests.

## Authors’ contributions

RZ and MS designed the study, MW carried out the elicitor treatments and the metabolite and molecular analyses, RZ carried out the molecular and genetic studies, RZ wrote the manuscript. All authors read and approved the final manuscript.

## Supplementary Material

Additional file 1 Individual glucosinolate content. Click here for file
