# Supplementary material for: Functional identification of genes responsible for the biosynthesis of 1-methoxy-indol-3-ylmethyl-glucosinolate in Brassica rapa ssp. chinensis
Source: BMC Plant Biol. 2014 May 8;14:124. doi: 10.1186/1471-2229-14-124 (PMC4108037; doi:10.1186/1471-2229-14-124)
Supplement: Additional file 1 — Individual glucosinolate content. [file 1471-2229-14-124-S1.pdf]

Table S1. Levels (µmol g^{-1} dry weight) of individual glucosinolates in sprouts of pak choi (*Brassica rapa* ssp. *chinensis*) 48 hours after application of different concentrations of methyl jasmonate (MeJA, µmol l^{-1}).

Glucosinolate	control	100	200	750	1,000	2,000	3,000
2OH3Ben	15.57 ± 1.07	19.62 ± 0.55	17.12 ± 0.37	18.22 ± 0.70	19.81 ± 1.24	24.08 ± 1.08	21.36 ± 1.28
4MSOB	0.27 ± 0.09	0.41 ± 0.08	0.37 ± 0.08	0.35 ± 0.07	0.46 ± 0.03	0.47 ± 0.04	0.41 ± 0.02
2OH4Pen	1.23 ± 0.11	1.82 ± 0.09	1.70 ± 0.15	1.84 ± 0.07	2.41 ± 0.11	3.06 ± 0.18	2.84 ± 0.14
3Ben	10.34 ± 0.20	10.12 ± 1.15	8.01 ± 0.98	6.56 ± 0.31	8.05 ± 1.16	7.64 ± 0.33	5.66 ± 0.90
4Pen	5.06 ± 0.14	5.19 ± 0.52	4.09 ± 0.51	3.60 ± 0.22	4.41 ± 0.60	4.45 ± 0.17	3.35 ± 0.58
4MTB	0.6 ± 0.69	0.67 ± 0.78	0.58 ± 0.68	0.17 ± 0.34	0.33 ± 0.38	0.42 ± 0.49	0.31 ± 0.36
2PE	0.21 ± 0.04	0.24 ± 0.09	0.17 ± 0.10	0.53 ± 0.07	0.62 ± 0.07	0.44 ± 0.51	0.15 ± 0.17
I3M	0.34 ± 0.02	0.89 ± 0.08	0.79 ± 0.04	0.78 ± 0.02	0.99 ± 0.11	1.08 ± 0.07	0.98 ± 0.15
4OHI3M	0.06 ± 0.05	0.07 ± 0.06	0.06 ± 0.05	0.05 ± 0.03	0.08 ± 0.03	0.07 ± 0.02	0.03 ± 0.03
4MOI3M	0.30 ± 0.02	0.37 ± 0.04	0.32 ± 0.05	0.35 ± 0.07	0.35 ± 0.05	0.44 ± 0.09	0.33 ± 0.04
1MOI3M	0.34 ± 0.04	2.98 ± 0.31	3.84 ± 0.65	6.27 ± 0.34	7.90 ± 0.44	10.36 ± 1.49	9.12 ± 1.67

2OH3Ben, 2-hydroxy-3-butenyl glucosinolate; 4MSOB, 4-methylsulfinylbutyl glucosinolate; 2OH4Pen, 2-hydroxy-4-pentenyl glucosinolate;3Ben, 3-butenyl glucosinolate; 4Pen, 4-pentenyl glucosinolate; 4MTB, 4-methylthiobutyl glucosinolate; 2PE, 2-phenylethyl glucosinolate; I3M, indol-3-ylmethyl glucosinolate; 4MOI3M, 4-methoxy-indol-3-ylmethyl glucosinolate; 1MOI3M, 1-methoxy-indol-3-ylmethyl glucosinolate

Table S2. Levels (µmol g^{-1} dry weight) of indole glucosinolates in sprouts of *Arabidopsis thaliana* 48 hours after application of different concentrations of methyl jasmonate (MeJA, µmol l^{-1})

Glucosinolate	control	200	control	500	control	5,000
I3M	3.08 ± 0.30	3.40 ± 0.31	1.24 ± 0.09	1.76 ± 0.12	1.25 ± 0.06	5.94 ± 0.42
4MOI3M	0.39 ± 0.07	0.50 ± 0.04	1.18 ± 0.11	1.28 ± 0.07	0.84 ± 0.03	1.04 ± 0.12
1MOI3M	0.25 ± 0.03	0.35 ± 0.07	0.24 ± 0.06	0.38 ± 0.03	0.16 ± 0.06	0.26 ± 0.04

I3M, indol-3-ylmethyl glucosinolate; 4MOI3M, 4-methoxy-indol-3-ylmethyl glucosinolate; 1MOI3M, 1-methoxy-indol-3-ylmethyl glucosinolate

Table S3. Levels (µmol g^{-1} dry weight) of individual glucosinolates in *Arabidopsis thaliana* tissue used as respective control in Table 4.

Glucosinolate	leaves	roots	leaves flowering plant
3MSOP	0.43 ± 0.01	-	0.17 ± 0.13
4MSOB	3.61 ± 0.02	-	0.96 ± 0.06
4MTB	3.65 ± 0.41	-	-
5MSOP	0.31 ± 0.01	-	-
8MSOO	0.68 ± 0.05	1.68 ± 0.00	-
I3M	1.59 ± 0.20	2.72 ± 0.05	1.13 ± 0.53
4MOI3M	0.91 ± 0.06	1.01 ± 0.11	0.20 ± 0.10
1MOI3M	0.24 ± 0.00	1.07 ± 0.27	0.18 ± 0.09

3MSOP, 3-methylsulfinyl-propyl GS; 4MSOB, 4-methylsulfinyl-butyl GS; 4MTB, 4-methylthio-butyl GS; 5MSOP, 5-methylsulfinyl-pentyl GS; 8MSOO, 8-methylsulfinyl-octyl GS; I3M, indol-3-ylmethyl GS; 4MOI3M, 4-methoxy-indol-3-ylmethyl GS; 1MOI3M, 1-methoxy-indol-3-ylmethyl GS. Values represent the mean ± standard deviation of three to six individual plants. -, below detection limit.
